# Fresh Air in Public Buildings

**Published:** 1906-12

**Authors:** 


### Abstracts and Selections

### Fresh Air in Public Buildings.

At the first thought it would seem that the President of the International Congress on Tuberculosis was putting it rather strongly when, in speaking of the transmission of consumption, he referred to the danger lurking in public buildings not properly cared for, and described churches as "lively disease breeders," and as "veritable Black Holes of Calcutta." The opinion changes, however, when it is realized that Dr. Daniel was basing his assertion on a definite calculation, and not upon a glittering generality. "I attended a church," he is reported to have said, "where, according to my calculation, the same air was breathed and rebreathed by each of 500 members of the congregation every ten minutes, or twelve times in the course of the service." In spite of the improvements architecturally and structurally in buildings of every class, it has to be confessed that practicable systems of ventilation are more conspicuous by their absence than by their presence, even in the newer structures, while they are altogether lacking in those of any age. That not only churches—and it is to be presumed that the older and less scientifically constructed buildings were those upon which the strictures were placed by Dr. Daniel—but practically all public gathering places in which large audiences congregate do not prove to be genuine Black Holes, veritable death traps, is due to the comparatively short periods during which they are occupied. And what is true of places of public assemblage is almost equally true of school buildings, of factories, of office buildings and of other structures that are occupied for hours at a time. Fortunately, in some of these temporary relief is possible through the raising or lowering of windows, without resort to the mechanical expedients that are required to secure a really adequate supply of fresh air for a large hall. But, unfortunately, the tendency toward overheating dwellings and offices and workshops, for which modern heating appliances are responsible, is turning the American of today into a tender house plant, that "catches cold" at the first puff of outside air.

The scientist is able to show by convincing experiment just to what extent the air is devitalized that is breathed over and over again, whether in public halls or in private dwellings. But the lassitude and weariness, the fatigue and inertia that follow an experience in a devitalized atmosphere are convincing enough without scientific demonstration. The only trouble is that the layman is apt to attribute them to mental or physical effort instead of to the real cause. The lowered vitality, and with it the decreased power of resistance to disease, which this rebreathing of vitiated air produces, is held by Dr. Daniel to be a prolific cause of consumption. And the white plague is only one of the communicable diseases of which vitiated air is a carrier. Fortunately, fresh air is not a luxury, but is easily obtainable. Like the majority of everyday blessings, its value it not always appreciated, and anybody who, like the President of the International Congress on Tuberculosis, directs attention to what its absence really portends, is doing a public service.—*American, Baltimore, Md.*

---

AN EDITOR was sitting in his office one day when a man came in whose brow was clothed with thunder. Fiercely seizing a chair, he slammed his hat on the table, hurled his umbrella on the floor, and sat down.

"Are you the editor?" he asked.

"Yes."

"Can you read writing?"

"Of course."

"Read that, then," he said, thrusting at the editor an envelope with an inscription on it.

"B—," said the editor, trying to spell it.

"That's not a 'B,' it's an 'S,' " said the man.

" 'S?' Oh, yes; I see. Well, it looks like 'Soles or dinner,' or 'Souls for sinners,' said the editor.

"No, sir," replied the man, "nothing of the sort. That's my name—Samuel Bruner. I knew you couldn't read. I called to see about that poem of mine you printed the other day entitled 'The Surcease of Sorrow.' "

'Souls for sinners,' " said the editor.

"Of course you don't, because it went into the paper under the villainous title of 'Smearcase Tomorrow."

"A blunder of the compositor, I suppose."

"Yes, sir; and that is what I am here to see you about. The way in which that poem was mutilated was simply scandalous. I haven't slept a night since. It exposed me to derision. People think me a fool. (The editor coughed.) Let me show you. This first line, when I wrote it, read in this way: 'Lying by a weeping willow, underneath a gentle slope.' That is beautiful and poetic. Now, how did your vile sheet represent it to the public: 'Lying to a weeping widow, I induced her to elope.' 'Weeping widow!' mind you. A widow! Oh, thunder and lightning! This is too much."

"It's hard, sir, very hard," said the editor.

"Then take the fifth verse. In the original manuscript it said, plain as daylight, 'Take away the jingling money, it is only glittering dross.' In its printed form you make me say, 'Take away the tingling honey; put some flies in for the boss.' By George! I feel like attacking somebody with your fire shovel! But oh, look at the sixth verse. I wrote, 'I'm weary of the tossing of the ocean as it heaves.' When I opened your paper and saw the lines transformed into 'I'm wearing out my trousers till they are open at the knees,' I thought that was taking it an inch too far. I fancy I have a right to murder that compositor. Where is he?"

"He is out just now," said the editor. "Come in tomorrow."

"I will," said the poet, "and I will come armed."—*Chicago Record-Herald.*

